# Colonial Virus”: COVID-19, creative arts and public health communication in Ghana

**DOI:** 10.4314/gmj.v54i4s.13

**Published:** 2020-12

**Authors:** Ama de-Graft Aikins, Bernard Akoi-Jackson

**Affiliations:** 1 Institute of Advanced Studies, University College London, Gower Street, London WCIE 6BT; 2 Department of Painting & Sculpture, Faculty of Art, College of Art and Built Environment, Kwame Nkrumah University of Science and Technology, Kumasi, Ghana

**Keywords:** COVID-19, creative arts, public health communication, behaviour change, Ghana

## Abstract

Since March 2020, Ghana's creative arts communities have tracked the complex facets of the COVID-19 pandemic through various art forms. This paper reports a study that analysed selected ‘COVID art forms’ through arts and health and critical health psychology frameworks. Art forms produced between March and July 2020, and available in the public sphere - traditional media, social media and public spaces - were collated. The data consisted of comedy, cartoons, songs, murals and textile designs. Three key functions emerged from analysis: health promotion (comedy, cartoons, songs); disease prevention (masks); and improving the aesthetics of the healthcare environment (murals). Textile designs performed broader socio-cultural functions of memorialising and political advocacy. Similar to earlier HIV/AIDS and Ebola arts interventions in other African countries, these Ghanaian COVID art forms translated public health information on COVID-19 in ways that connected emotionally, created social awareness and improved public understanding. However, some art forms had limitations: for example, songs that edutained using fear-based strategies or promoting conspiracy theories on the origins and treatment of COVID-19, and state-sponsored visual art that represented public health messaging decoupled from socio-economic barriers to health protection. These were likely to undermine the public health communication goals of behaviour modification. We outline concrete approaches to incorporate creative arts into COVID-19 public health interventions and post-pandemic health systems strengthening in Ghana.

**Funding:**

None declared

## Introduction

“Herh, School Boy, Timothy bra bra. Ei, Timothy ɛtesɛn?

Timothy: Mepawokyɛw ɛyɛ oo

Aha, Timothy ka corona ABCD no kyerɛ me

Timothy: Hehe, Colonial ABCD

A: Abroso ooo, abɔ yɛ so! ama afei yɛ hyehyɛ dan mu

B: Bibiara ɛnnyɛ me ehi sɛ, yɛ bɔ wa dada nɛnso sɛ ɛnɛ, ntɛsuo twi wo koraa a, nna obi pɛ wo abɔ wo dua.

C: Colona vilus, Nyame betua wo ka.

D: Didi yie ooo menua Kwame, didi yie na fa vitamin A, B, C, D, E, F…”

“Hey, School boy, Timothy, come, come here. Ei Timothy how are you?

Timothy: Please, I'm fine oh

Aha, Timothy, tell me the corona ABCD

Timothy: Hehe, Colonial ABCD

A: It is so overwhelming, we are in shock! Now, we are all hiding indoors.

B: Nothing annoys me more than the fact that we have always had our coughs. But now, even when you choke on your saliva, somebody wants to curse you.

C: Colona vilus, God will punish you

D: Eat well ooo my brother Kwame. Eat well and take vitamins A, B, C, D, E, F…”

(“Corona ABCD” by Clemento Suarez Source: https://www.facebook.com/watch/?v=1369509386567639.

Transcription and translation from the original Twi by Jemima Okai, Francis Agyei and Ama de-Graft Aikins)

This exchange came from a comedy video that went viral on social media in March 2020, just days after lockdown was imposed on Accra, Kumasi and selected communities. The video featured the Ghanaian comedian and artist, Clemento Suarez (real name Clement Ashitey), playing a schoolboy called Timothy who was walking along the road in the standard brown and beige school uniform, when he was stopped by a female journalist. The journalist, in the style of a media vox pop, asked him to recite “the Corona ABCD”. As Timothy worked his way through the ABCDs of ‘colonial’ virus, he told a funny, but complex, story of the impact of the COVID-19 pandemic in Ghana. He touched on public health, politics, religion and economics, as well as collective memories of past national hardships, such as the 1983 famine and the curfews of the 1970s coup years. Watching the video, Suarez made you laugh and think.

Suarez's comic video joins a growing trend of responses to the COVID-19 pandemic by Ghana's creative arts communities, since the first two cases were reported on 12^th^ March 2020. Whether produced by known or unknown artists, self-funded or state sponsored, their art forms track the complex facets of the pandemic.

In this paper we review representations of COVID-19 by creative art communities in Ghana between March and July 2020. We have two aims: (1) to describe the types of creative arts representations and their functions; and (2) discuss how these art forms offer important models of public health communication that can shape public health interventions during the COVID-19 pandemic and beyond.

### Conceptual framework

The arts serve multiple functions in Ghanaian socio-cultural life. They are incorporated into significant cultural rites of passage, from the outdooring of new births and marking of puberty, to the mourning of death. They mediate cultural reflection, critique and knowledge production.^1^ They support political and religious systems and practices.^2^ The arts are also integral to health and healing. Traditional healing systems in Ghanaian communities have incorporated the arts, through the use of objects, artefacts, costumes, singing, drumming and theatre. The arts have also been incorporated into contemporary public health promotion and interventions, from the earliest posters educating on childhood diseases to the billboards, radio jingles and television dramas on public health crises and recurrent health threats today.^3^,^4^

The field of arts and health provides a useful framework to conduct a systematic analysis of the role of arts in Ghana's COVID-19 response. Health psychologists define the field as one that integrates the arts into “health promotion, disease prevention, policy development, illness management and aesthetics of the healthcare environment” (p.288).^5^

In Africa, the field has focused predominantly on HIV/AIDS education, care and empowerment, although newer themes, such as maternal health and stroke, have emerged in the last decade.^6^ In Ghana, visual art, music, dance, storytelling, theatre/drama have been applied to explore community knowledge and responses to HIV/AIDS, malaria, cholera, mental illness, as well as addressing general health and wellbeing.^7^–^9^ Studies show that in Ghana, as reported elsewhere, the arts are useful tools for health communication and healthcare provision. The studies also highlight fundamental barriers to meaningful health education and equitable healthcare, such as the predominant use of English language in health communication and the exclusion of lay perspectives in the development of health interventions and healthcare services.

We examine COVID art forms and their functions within this arts and health framework, and from a critical health psychology perspective. Critical health psychology, as practiced globally and in Ghana, adopts a multilevel approach that theorizes the individual, social, cultural, economic and political contexts of health and illness, applies context specific methods, and addresses problematic expert assumptions, ideologies and systems.^5^,^10^ Critical health psychology research has contributed to the interdisciplinary field of public health communication, which aims to facilitate optimal public health through behaviour change at multiple levels of social organization: individual, group, community and societal.^11^

## Methods

We collated data available in the public sphere between March and July 2020. We defined ‘COVID art forms’ as any type of artistic expression that incorporated themes on the COVID-19 pandemic. We included visual art, music, comedy, dance, theatre, literature and textile design into an open-ended range of artistic expression – these forms could be contemporary or traditional, popular or elite. COVID themes included public health terms (e.g. ‘social distancing’, ‘test and trace’), political terms (e.g. ‘COVID relief fund’, ‘government directives’), economic terms (e.g. ‘COVID-19 economy’) and socio-cultural translations or “localized terminologies”^6^ of COVID-19 scientific or medical concepts (e.g. ‘colonial virus’ instead of coronavirus). Using a rapid appraisal strategy, we tracked social media (Facebook, Instagram, Twitter, YouTube, WhatsApp), reviewed newspapers for cartoons and commentary, and solicited additional material through our Ghanaian social and creative arts networks. Table 1 presents a summary of the collated data.

**Table 1:** Art forms and data sources

COVID-19 art form	Source(s)
**Comedy skits**	Social media: WhatsApp, Facebook
**Songs**	Social media: WhatsApp, Facebook
**Cartoons**	Traditional media: Daily Guide; Business & Financial Times. Social media: Facebook
**Murals**	Creative arts network
**Fashion**	Social media: WhatsApp, Facebook, Instagram Social networks and public spaces
**Textile** **designs**	Social media: WhatsApp, Facebook, Instagram, Twitter, Traditional media: television, radio

The examples we use here are published or have traceable sources that ensure we maintain ethical standards, but also allow for replication and follow up studies.

## Results

We provide contextual descriptions and functions of the six COVID art forms presented in Table 1. Because textile designs and fashion intersect in production and function, we treat both under one section. We describe each art form and discuss its functions within the ‘arts and health’ framework. We also highlight strengths and limitations, signposting these for further discussion in the following sections.

### Comedy, laughter and cultural critique

The vox pop (also vox populi) is referred to as the “voice of the people” or “the opinion of the majority of the people”.^12^ This method is used by the major Ghanaian media networks to gather on-the-spot views and perspectives from the public. Before the COVID-19 pandemic, typical questions would focus on topical issues or controversies of the day, such as lay views on Ghana's Independence during Independence Day, or specific cases of political corruption. Part serious, part played for laughs, but always multilingual, vox pops educate the Ghanaian public on what is widely known and understood, or what might be an emerging issue of public interest. The most hilarious encounters are often turned into memes or gifs and shared by thousands in Ghana and the Ghanaian diaspora via social media, creating - willing or reluctant - social media stars.

Suarez's comedy skit on the ‘corona ABCD’ falls under the broad category of comedy and the specific sub-category of the parody vox pop. Even before the Suarez video went viral, there had been vox pops involving journalists engaging market women, tro-tro drivers, school children, *kayayei*, and members of poor urban and rural communities in COVID narratives. These were broadcast on major television and radio stations, as news segments. The comedic versions reworked the insights from the media vox pops and inserted new humorous dimensions – and circulated via YouTube, Facebook or WhatsApp.

The media vox pops and the comedic versions illuminated lay understandings and misunderstandings of the seriousness of the pandemic and of preventive measures. In the early months of March and April, rumours spread that the local brew *akpeteshie* could be used as a hand sanitiser, as well as a sedative to ease the stresses of lockdown (see the section on cartoons). In some communities, both urban and rural, people were sceptical that the virus existed or was deadly.

But the vox pops and comedy skits also tapped into the Ghanaian collective sense of humour. Before the World Health Organization's (WHO) declaration of COVID-19 as a global pandemic on 11^th^ March, ‘localized terminologies’ of the pandemic were circulating in social networks and social media. These covered the name of the virus itself (e.g. “colonial virus”, “corona” “colonsa vilus”), hand sanitizers (e.g “ante antelizer”), social distancing (e.g. humorous alternatives to handshakes, such as the elbow shakes and foot shakes), mask wearing and other aspects of the pandemic. Between March and July the president gave regular national addresses to the nation detailing official COVID response strategies and progress. His trademark greeting of “Fellow Ghanaians”, and other repeated phrases in his addresses (e.g. “The battle is the Lord's”) entered the social lexicon of popular greetings, jokes and satire. Recent iterations of COVID jokes include naming children during this pandemic era, for example Akosua Sanitiser and Kwaku Lockdown.

Barz and Cohen^6^ observe that “music as medical intervention” worked for AIDS in Uganda because: “when technical, scientific, or medical ‘AIDS talk’ was abandoned in favour of “un-translated” localized terminologies” … “audiences appeared much less threatened and anxious”. (p.8) …Heads nod in agreement or hands are clapped in laughter when particular lines resonate with the audience's experience” (p.10).

By using humour and eliciting laughter, comedy, like music, can act “as medical intervention”. When Suarez uses the term ‘colonial virus’ in his video he taps into ‘localized terminologies’ of COVID, as well as the Ghanaian collective sense of humour. While these terminologies function as a comedy device, they also function asa route to health education. His audience laughs, they listen on and then, may be more open to absorbing new ideas and information.

### Cartoons and socio-political commentary

We collated 53 cartoons produced by three cartoonists between March and July 31^st^, 2020. The cartoonists draw under the artistic names of Akosua, Makaveli and Tilapia da Cartoon. Akosua and Makaveli draw for the Daily Guide and Business and Financial Times, respectively. Tilapia da Cartoon publishes on Facebook. In Table 2, we present a thematic summary of the cartoons and selected titles from each artist. Collectively, the cartoons tackled social, economic, public health, religious and political themes: about a third of the cartoons tackled blended themes.

**Table 2:** Cartoon themes and selected titles

Themes	Number of cartoons	Selected titles (by cartoonists)
**Social**	6	“Covid-19: greetings change” (Tilapia, 12^th^ March) “Apio Sanitizer” (Akosua, 17^th^ March)
**Economic**	4	“Balanced diet versus balancing empty pockets” (Makaveli, 20^th^ April) “COVID-19 economy versus Dumsor economy” (Tilapia, 7^th^ May)
**Public health**	19	“social distancing” (Akosua, 27^th^ March) “rise in COVID-19 cases” (Makaveli, 12^th^ May) “COVID-19 Figures Massage Centre” (Tilapia, 17^th^ June)
**Religious**	2	“Church online” (Akosua, 19^th^ March)
**Political**	7	“Parliamentary corona waters” (Tilapia, 29^th^ May)
**Blended themes**	12	“Oga! Please where is the $100 million” (Tilapia, 20^th^ March) “food sharing and social distancing wahala” (Makaveli, 15^th^ April) “face mask and voter registration” (Akosua, 9^th^ July)

**Figure 1:**
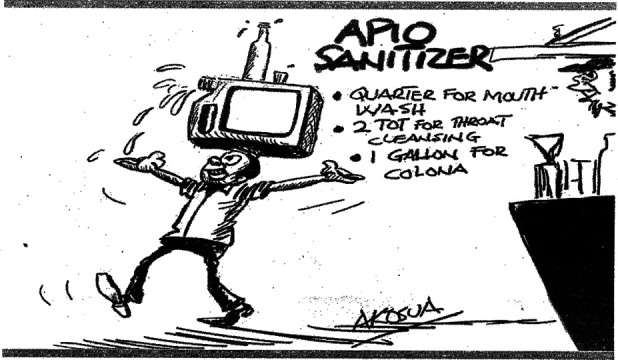
“Apio sanitizer”, Akosua, Daily Guide, 17^th^ March 2020

**Figure 2:**
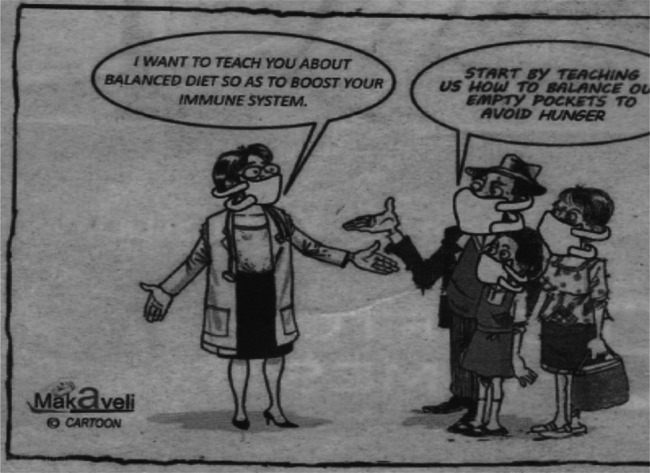
“Balanced diet versus balancing empty pockets” Makaveli, B&FT, 20^th^ April 2020

**Figure 3:**
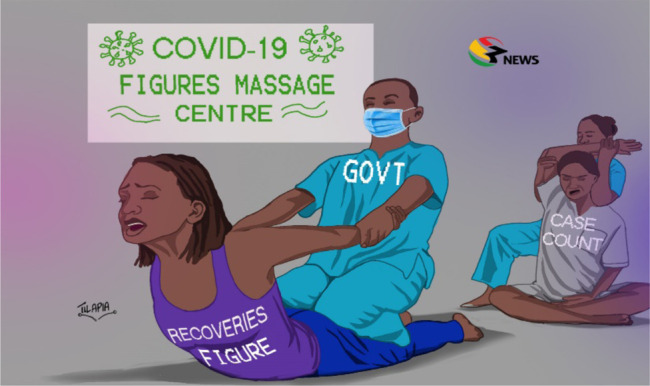
COVID-19 Figures Massage Centre, Tilapia, Facebook, 17^th^ June 2020.

We thematised the cartoons based on their titles and visual representations. Social themes focused on social understandings and responses to the pandemic (see Figure 1). Economics focused on the economic impact of the pandemic on the nation and on ‘vulnerable communities’ - defined in an early presidential address as poor and marginalised communities who were predicted to experience a disproportionate impact of the pandemic (see Figure 2). Public health themes covered prevention, testing and associated aspects of health systems responses (see Figure 3). Religious themes focused on the responses to the pandemic by religious institutions and communities. Political themes focused on responses by government, politicians and policymakers.

Some cartoons featured blended themes. For instance, Makaveli's April 15^th^ cartoon titled “food sharing and social distancing wahala” blended the political theme of COVID relief (implementing the policy of food distribution to vulnerable communities) with public health theme (of social distancing). Similarly, Akosua's July 9^th^ cartoon titled “face mask and voter registration” highlighted the complications of navigating voter registration (politics) and COVID prevention (public health).

While each artist employed a different artistic style, in terms of drawing and use of colour, they all blended standard reporting of trends in the national COVID response and the genre of political cartooning. The genre incorporates “allusions, satire and innuendos in depictions of certain (political) personalities and situations” (Oduro-Frimpong, 2014, p.135).^13^

The cartoonists, like the comedians, also blended local languages, localized COVID terminologies, pidgin English and standard English: ‘apio’, ‘tot’, ‘Oga’ ‘colona’ ‘wahala’ and ‘palava’. These communicative strategies were particularly obvious on the themes that spoke to complex socio-political challenges and public anticipation of institutional corruption: inequitable food distribution to poor and vulnerable communities, disputed COVID-19 statistics generated by the Ghana Health Service, and the potential misdirection of COVID-19 funds - in particular the nationally generated COVID-19 alleviation fund and the USD$1 Billion COVID relief loan from the International Monetary Fund (IMF) – for political purposes (see Table 2).

‘Allusions, satire and innuendo’ are established communicative strategies through which the traditional arts critique cultural systems. Across Ghanaian cultures, folklore, folk songs, and theatre, for example, have used satire and subversive critique to challenge social norms and authority, and confront taboos and sacred institutions.^1^

Whether formally educated or not, most Ghanaians recognise these communicative strategies, particularly when they are presented visually. When cartoonists use subversive strategies of political cartooning to tell the evolving story of the pandemic, they do not only reflect the fears and anxieties of the public back to powerful political actors and institutions. They also conscientize their audiences to the inconsistences and hypocrisies of political systems and power, through a shared cultural language. And when audiences are large, connected and engaged, for instance on social media, the critique grows in moral power and can lead to collective action for change.

### Songs and COVID edutainment

The fourteen songs we collated (see Table 3) fell under the category of “edutainment”. Edutainment has been defined as “the process of purposely designing and implementing a media message to both entertain and educate, in order to increase knowledge about an issue, create favourable attitudes and change overt behaviour” (Alviso, 2011, p.57).^14^ All the songs used catchy melodies, hooks or choruses, and lyrics in Ghanaian languages (Ahanta, Ewe, Fante, Ga, Twi), in pidgin English, or blended English and Ghanaian languages.

**Table 3:** COVID song genres, artists and titles

Genre	No of songs	Language	Titles and artists
**Afro-pop**	1	English	“Coromental” (Reggie n Bollie)
**Folk**	2	Twi, Ga, Ewe	“COVID-19” (Abibiman, performed by Abena Owusua & Kokofu Serwaa) “Corona Virus” (School Children)
**Gospel**	2	Twi	“Corona Virus” (Ama Grace) “Corona Virus” (Great Ampong)
**Highlife**	2	Ahanta, Ewe, Fante, Ga, Twi, Pidgin English	“Corona” (Abochi featuring Dede Supa) “Corona Virus” (Kofi Kinaata)
**Hiplife/hiphop/rap/trap**	6	Twi, Ga, English	“Corona Virus” (Cryme Officer) “Stay Home, Stay Safe” (Naa Ashorkor, Clemento Suarez and Deelaw) “Coronavirus freestyle” (Opanka) “Corona Virus” (Patapaa Amisty) “Corona” (Tulenkey)
**Reggae/Dancehall**	2	Twi, English	“Corona Virus” (Article Wan) “Corona Virus” (Bless)

All the songs focused on public health prevention aspects of COVID, such as handwashing, respiratory hygiene and physical distancing. Some outlined the symptoms and medical outcomes of viral infection such as coughing, fever and loss of smell and taste. Most of the songs also incorporated additional social, economic, religious and political themes into their public health messaging. The social theme described the widespread impact of COVID across social class and status (‘rich’, ‘poor’, ‘Kings’, ‘Chiefs’) and professions (‘doctors’, ‘lawyers’, ‘soldiers’, ‘pastors’ ‘mallams’) across the country. Gospel artists foregrounded the religious and spiritual dimensions of healing in their lyrics, as is expected for the genre. But other genres also incorporated spiritual/religious features, by invoking God's protection in the fight against the virus or observing the impact of the pandemic on church activities. Hiplife/hip hop artists focused on the global and national politics of pandemic control. Some pushed conspiracy theories on the origins of COVID-19 within this broader framework.

The hiplife track (“Corana”) by Tulenkey, included a verse on the negative economic impact of the pandemic on artists' livelihoods. This subject became widely discussed in the performing arts community during the period under analysis. The highlife song (“Corona Virus”) by another artist, Kofi Kinaata, targeted the lives and livelihoods of Ewe, Ga, Fante and Ahanta fishing communities. His song - and accompanying video - was sponsored by state ministries, national fishing organizations and development partners, namely: Ministry of Fisheries and Aquaculture Development, Fisheries Commission, Ghana National Canoe Fishermen Council, National Fish Processors and Traders Association, University of Rhode Island and USAID.

Three types of edutainment that emerged in the analysis: the humorous, fear-based and conspiracy-based. Some songs blended the three approaches. Kofi Kinaata's song was a professionally produced version of the humorous edutainment approach. The song offered a comprehensive public health message presented in the highlife artist's humorous and satirical signature style, the memorable melody and chorus were laid over a dance track, and the music video featured working and dancing members of the target communities.

The song “COVID-19” produced by the Abibiman group was composed and sang in the folk song genre. The song had three verses. The first verse covered the core themes of COVID prevention: social/physical distancing, avoiding handshakes, handwashing, respiratory hygiene, and avoiding COVID hotspots. The second verse highlighted the psychological and medical impact of the virus. The third verse detailed the non-discriminatory impact of the virus on “Kings, Chiefs, Pastors, Traditional priests, mallams, the rich and the poor”. In the style of folk songs that seek to entertain, educate or edutain, the Abibiman song used repetition of selected lines to emphasise the core message:

**Table:** 

Corona virus akoadiɛ wuo yi ɛnya wo aa ɛbe kumwo ɛde wo bɛkɔ asaman akyere do oooo	Coronavirus, that deadly disease When it gets you, it will kill you It will take you to the land of the dead
Yeee covid-19 kɔdea wuo yi Abasen yɛn koomu yi Yadea yi ɛnya wo aa ɛbɛ ha wo Yadea yi ɛnya wo aa ebeku wo	Yeee this deadly covid-19 That has befallen us When this disease gets you, it will disturb you When this disease gets you, it will kill you

(Transcription and translation from Twi by Jemima Okai, Francis Agyei and Ama de-Graft Aikins)

These repeated lines emphasised the destructive and deadly impact of the coronavirus: “it will disturb you”, “it will kill you”, “it will take you to the land of the dead”. The overall tone of the song took on an authoritative expert voice that invoked fear in order to enforce COVID prevention behaviour: if listeners did not take the advice, they were likely to encounter destructive social and psychological hardship or die from viral infection.

In two hip life songs, “Corona (Freestyle)” by Opanka and “Corona” by Tulenkey, both artists emphasised the importance of prevention, placed the local reality of COVID in Ghana in global perspective and raised concerns about the economics and politics of pandemic control. However, the political sub-themes were framed in conspiracy theories about the Western (‘Whiteman’) origins of COVID and Western medical interventions for COVID. Like the localized terminologies used by comedians and cartoonists, these conspiracy theories were reworked ideas circulating physical and online Ghanaian and African social networks. We present excerpts of Tulenkey's song performed in a mix of pidgin English and Twi.

**Table:** 

“Yɛte hɔ aa mo se Bird Flu (bird flu) Ankyɛ na mo se Swine Flu (swine flu) Yɛbɛ te y'ɛni aa rabies (rabies) Yɛda ne yɛn ho HIV (mokoraa adɛn!) Afei na corona (rona) Saa na mo yɛɛ Ebola (bola)	At first you all said Bird Flu (bird flu) Soon after you said Swine Flu (swine flu) Before we realised it was rabies (rabies) We turned around, HIV (what is it with you all!) Right now it is corona (rona) Same way you made Ebola (bola)
White man always tryna find ways to eradicate black population Oh no Leave the Black alone Why do you kill your own? Global war but everybody jie eye^1^ dey protect their own” *(“Corona” by Tulenkey. Transcribed by Jemima Okai, Twi translation by Ama de-Graft Aikins)*	

While the humorous edutainment approach offered ‘music as medical intervention’^6^, the fear-based and conspiracy-based edutainment approaches undermined the goals of health education and promotion. By mixing the facts of COVID prevention with conspiracy theories about origin and treatment, the hiplife songs fed into ‘infodemics’ 15 - the blend of misinformation and disinformation on public health crises that is amplified on social media and undermines health protective behaviours. Some Ghanaian musicians and pop artists have a powerful online presence (see Appendix 1). Therefore, what they produce and disseminate on COVID matters to Ghana's pandemic health policy.

### Murals and improving ‘aesthetics of healthcare environments’

In March, the Ghana Graffiti Collective painted a mural on COVID prevention in a suburb of Accra. Their work was supported by the Accra Metropolitan Assembly (AMA), the International Organization for Migration (IOM), and the Delegation of the European Union in Ghana.

The mural falls under the category of artists-state collaborative projects. In recent years of mural and graffiti artists have received support from arts organizations and collectives such as the Ghana Association of Visual Artists, Ghana Graffiti Collective and Accra Dot Alt, as well as from state and development partners on projects aiming to transform the aesthetics of public spaces. The COVID mural also joins collaborations between artists, the state and development partners on public health, such as campaigns on HIV/AIDS prevention in the 1980s and 1990s,^3^ the National Truly Clean Hands campaign in 2003,^4^ and the Kofi Kinaata Coronavirus song for fishing communities.

The mural featured the key COVID prevention strategies of handwashing, respiratory hygiene and face mask wearing. Like the COVID songs, the function of the mural was to educate the public, who drove or walked past, about the virus. It replaced the typical eclectic collection of posters, flyers and graffiti on public walls, with a panoramic visual image and story in vivid colours - yellows, blues, reds and purples - that caught the attention and lingered in the subconscious long after one had driven or walked past. The health subject matter and its psychodynamic impact on observers, therefore, also functioned to transform the aesthetics of the health environment.

Like the COVID songs, the mural offered a didactic approach to health promotion. The public was educated on COVID prevention, but the complex determinants of health prevention and illness management were not addressed. For example, one prominent image in the series was of a tap with running water under which a woman washed her hands with a bar of soap. This image suggested that everyone has access to water, when this smoothed over the complicated reality of water poverty. The vast majority of rural Ghanaians do not have pipeborn or safe potable water. And even in urban areas, where homes may have taps, and residents pay monthly water bills, the taps do not produce a gushing flow of water.

### Textile designs, fashion and memorialising the pandemic

Months before face masks became mandatory in June 2020, a creative cottage industry emerged on the production of wax print masks and of fashion matching clothing with masks of identical textile designs. Marketed on social media – Facebook, Whatsapp, Instagram, Twitter - these products reached thousands of potential and actual buyers beyond immediate physical social networks.

Making masks fashionable and fun facilitated the social adaptation of an uncomfortable but necessary new habit in Ghana, long before other countries like the UK and US that are still grappling with the politics and logistics of face coverings several months later. But it also created financial opportunities in a time of economic uncertainty for seamstresses and tailors, as well as more established designers. The grassroots response to local masks and ‘COVID fashion’ also appears to have informed a new government policy of investing in local production of face masks.

In July 2020, Ghana Textiles Printing (GTP), launched new COVID-19 inspired textile designs. The designs updated classic GTP wax print designs such as ‘*Aban kaba*’ and ‘*Ahenepa nkasa*’ with symbols representing significant aspects of the pandemic. These included planes, padlocks, the coronavirus, and in what appeared to be political homage, the president's distinctive round eyeglasses. The new designs were labelled “Fellow Ghanaians” and “Lockdown”, the former capturing the president's standard opening line in his addresses to the nation, the latter capturing the three-week period of partial lockdown in March and April.

In an interview with BBC Focus on Africa, the marketing director of GTP, Mr Stephen Badu, explained the motivation driving the designs:

"We are a business that tells stories, and we tell our stories through our designs. We believe that it [COVID] is going to leave a mark in the history of the world, and it's important that generations that come after us get to know that once upon a time, such a phenomenon occurred… Behind every design we produce it's our value systems, our sense of art, and how we communicate"^16^

This origin story told by the marketing director illuminated the cultural significance of textiles beyond their function as fashion. The GTP COVID designs built on an existing culture of producing new textile designs or choosing existing designs with appropriate meaning to commemorate significant social, community and family events or to brand institutions.

While the GTP design was produced for commercial profit, the designs memorialised a significant evolving event in global and national history. They operated in the same way as the Ghana@50 designs produced in 2007 to mark Ghana's 50^th^ anniversary of independence from British colonial rule. Within weeks of their launch, the new textile designs were used to clothe beauty queens in the annual televised Ghana's Most Beautiful pageant and incorporated into the 2020 election campaign paraphernalia by the incumbent New Patriotic Party (NPP) government.

## Discussion

This study had two aims: (1) to describe the types of creative arts representations and their functions; and (2) discuss how these art forms offer important models of health communication that can shape public health interventions during the COVID pandemic and beyond.

The featured art forms performed three key functions within the ‘arts and health’ framework: health promotion (comedy, cartoons, songs); disease prevention (masks); and improving the aesthetics of the healthcare environment (murals). While locally made masks offered protection from infection, textile designs and fashion generally, performed a broader socio-cultural function. By memorialising COVID as a significant public health and cultural event they contributed to the production of collective memory and the expansion of cultural knowledge on pandemics, public health threats and associated significant national events. GTP also expanded its purported role as a ‘storyteller’ into political advocacy, by paying political homage to the president in their COVID designs, and having their designs used in party political campaigning in an election year.

The Ghanaian COVID art forms are similar to previous HIV/AIDS and Ebola arts-based interventions in other African countries: they connect culturally and emotionally, and they offer important alternatives and additions to the official public health response to COVID. Artists' use of social media, in particular, means they reach wide audiences within and outside Ghana's borders.

But some of the art forms had limitations. We highlighted the folksong that seeks to edutain but uses fear appeals, the hip-life songs that feed into infodemics by pushing conspiracy theories on COVID origins and treatment, and the state-sponsored mural that represents public health messaging decoupled from the socio-economic barriers to health protection. These art forms might contribute to public understanding of COVID in the shortterm, but they are also likely to undermine sustained behaviour modification or transformation in the long-term. While coronavirus is indeed destructive and deadly, the fear approach to public health communication is not the most effective strategy. Fear is a complex emotion.

Under some circumstances, it might motivate change towards healthy behaviours. Under other circumstances, it might prevent such an outcome and instead lead to discomfort, resistance or apathy. Fear-based strategies did not work for early health promotion campaigns on dental hygiene, seat belt wearing, anti-smoking, safe sex and HIV prevention in several countries.

Critical health psychology research demonstrates that the complex and context-dependent functions of fear often lead to unpredictable collective and individual outcomes, particularly when the threat is abstract.^11^ Furthermore, the functions of fear must be understood within the historical context of cultural responses to unfamiliar health threats.^17^

At a cultural psychological level, the popular Ghanaian slogan of ‘all die be die’ structures casual attitudes to health, illness and dying particularly in poor and marginalised communities. The normalization of casual attitudes to death and dying, which blurs cultural distinctions between good death and bad death, has been forged by decades of structural neglect, and increasing rates of developmental and health problems in marginalised communities. But this collective attitude is also anchored within existing cultural responses to death and dying, where socio-cultural investments are made for the dead and family members who stand to benefit from donations and inheritance, rather than for the seriously sick and the family members burdened with care.^18^ These complex socio-psychological and cultural dynamics, undermined fear-based strategies used for HIV prevention in Ghana in the 1980s and 1990s.^19^ Against this cultural and historical background, health communication campaigns on COVID that use fear of death and dying as a motivational strategy are unlikely to achieve their goals.

A second argument made in critical health psychology is that where there are no enabling environments to support new health behaviours and habits, people are unlikely to adopt them. At the early stages of the pandemic, local experts highlighted several environmental barriers to COVID prevention, including the lack of safe running water in Ghanaian homes, the difficulties of social distancing in poor communities and crowded households, and the conflicts inherent in obeying lockdown measures under conditions of financial and nutritional insecurity. Without attention to these structural barriers, behaviour change is compromised. Makavelli's cartoon contrasting the public health message of eating a balanced diet with the reality of food insecurity in poor communities (see Figure 2), presented a clear example of this challenge.

Sonke and Pesata, writing on Ebola arts interventions in Liberia, Sierra Leone and Guinea, observe that “when people engage emotionally with *correct information* through the arts, they share that information with others, creating an organic and meaningful dissemination of knowledge” (emphasis added).^20^ Incorrect and misleading information can be shared as widely as medically correct information.

A key feature of infodemics is the way misinformation strikes an emotional chord and catalyses viral communication. In a public context where infodemics are prevalent, influential artists who push conspiracy theories on online platforms contribute to their spread and harmful public health effects.

Misinformation on vaccines is particularly problematic in the Ghanaian context. Vaccination resistance is a global phenomenon.^21^ But, in Ghana and across Africa, this socio-psychological response is complicated by a long history - dating from the colonial era - of mistrust and antagonism towards Western medical interventions, from blood work for public health research to vaccine trials.^22^ Tensions and controversies around the Ebola vaccine trials in Ghana are a recent case in point.^23^ We drew attention to the fact that the conspiracy theories promoted in the hiplife songs were reworked from lay theories already circulating in physical and online Ghanaian and African social networks. This is an area that requires critical attention and vigilance as Ghana moves into the phase of the pandemic, where the social, economic and psychological impact becomes more salient.

This paper has limitations. We did not interview artists and audiences who consume the COVID art forms described here. This was due largely to the ethical and health risks inherent in conducting face to face interviews during a period coinciding with lockdown, followed by physical distancing rules, as well as the documented stresses and constraints of conducting online interviews during the pandemic. Furthermore, our engagement with social networks and public spaces was limited to Accra and Kumasi. Incorporating the perspectives of artists and lay consumers of art beyond Accra and Kumasi would have strengthened our arguments and provided insights into additional environmental barriers to COVID prevention strategies, as future public health interventions will benefit from the broadest evidence base. Finally, while the featured artists have an extensive online presence and an engaged community of followers, a predominant focus on online communities excludes older and offline communities. How these communities access and engage with COVID art forms is also of research importance. These are themes we intend to explore in follow up studies. However, this is the first study examining how an arts and health approach can contribute to the public health communication on COVID-19 in Ghana. We have presented preliminary insights on useful models that can be developed in more critical directions during the pandemic and beyond.

## Conclusion

Official COVID-19 cases in Ghana have risen from the first two cases reported on 12^th^ March to 41,003 and 215 deaths (at the time of writing on 5^th^ August 2020).^24^ As the pandemic evolves and the broader social and economic impact intersects with the medical, public health and health systems responses will have to focus on longer-term impact and effects on COVID-19, such as health-worker burn-out, chronic care, and public mental health effects of the pandemic. The arts can contribute to immediate and longer-term public health efforts.

## Figures and Tables

**Figure 4:**
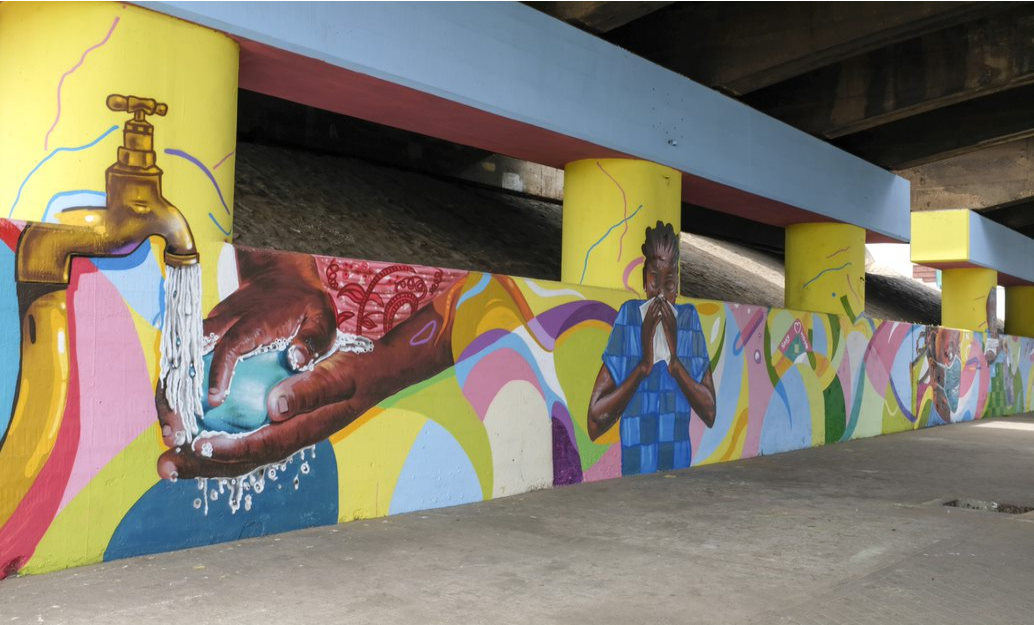
Mural on COVID-19 prevention produced by Ghana Graffiti Collective in collaboration with International Organization for Migration (IOM), Accra Metropolitan Assembly (AMA) and the Delegation of the European Union in Ghana. 2020. Photo: IOM.

**Figure 5:**
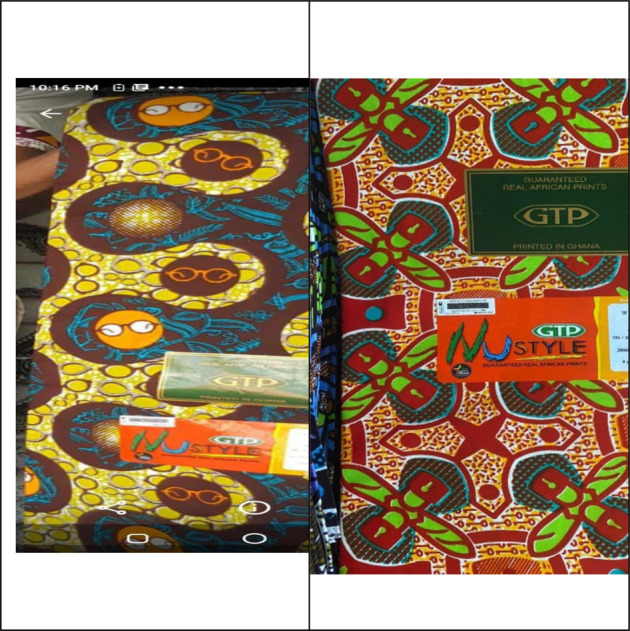
GTP “Fellow Ghanaians” (left) and “Lockdown” (right) NuStyle cloth. Printed by TexS (Photos by authors)

## Footnotes

1 “Jie eye”: Pidgin English for turn a blind eye, or ignore.

## Appendix 1: COVID Songs, Artists and Artists' Online Presence

**Table:**

	Song titles and sources	Artist(s)	Instagram	Facebook	Twitter	YouTube (Views; Subscribers - Subs)
**1.**	Coromental https://www.youtube.com/watch?v=D9-EeokV0I0	Reggie n Bollie	149 thousand	110,436	84.5 thousand	17 thousand views 4,660 subs
**2.**	COVID-19 https://www.youtube.com/watch?v=360mc1s-l5M	Abibiman (Abena Owusua & Kokofu Serwaa)	-	35	-	166 views 680 subs
**3**	Corona Virus Shared via WhatsApp groups.	School Children	n/a	n/a	n/a	n/a
**4.**	Corona Virus https://www.youtube.com/watch?v=88F-aajD0as	Ama Grace	51	3,858	-	19,000 views 2.2 thousand subs
**5**	Corona Virus https://www.youtube.com/watch?v=EduNrGL5OwE	Great Ampong	675	2,201	-	72 thousand views Subs are not public
**6.**	Corona https://www.youtube.com/watch?v=vrk8LrXLHys	Abochi ft Dede Supa	17.1 thousand	4,338	3,018 thousand	2.9 thousand views 50 subs
**7.**	Corona Virus https://www.youtube.com/watch?v=hygo8Xrn4qc	Kofi Kinaata	2 Million	490,685	266 thousand	266 thousand views 216 thousand subs
**8.**	Corona Virus https://www.youtube.com/watch?v=92eCk26UT9E	Cryme Officer	11 thousand	6,198	701	32 thousand views 74.8 thousand subs
**9.**	Stay Home, Stay Safe https://www.youtube.com/watch?v=vpVz-kwXd3w	Naa Ashorkor Clemento Suarez Deelaw Beatz	2.1 Million 897 thousand 582	47,347 18,156 -	39.6 thousand 34.5 thousand 154	12 thousand views 152 subs
**10.**	Coronavirus freestyle https://www.youtube.com/watch?v=6UKTW-usJ14	Opanka	359 thousand	452,112	71 thousand	2.3 thousand views Subs are not public
**11.**	Corona Virus https://www.youtube.com/watch?v=5AGR0PZxldA	Patapaa Amisty	559 thousand	5,975	43 thousand	69 thousand views 41.8 thousand subs
**12.**	Corona https://www.youtube.com/watch?v=FFFy8UNle0U	Tulenkey	192 thousand	37,195	101 thousand	98 thousand views 32, 600 subs
**13.**	Corona Virus https://www.youtube.com/watch?v=KLXXt4HGFw	Article Wan	81.3 thousand	33,115	5,564 thousand	3.3 thousand views 16,300 subs
**14.**	Corona Virus https://www.youtube.com/watch?v=U0rJmNRy6s0	Bless	25.8 thousand	69,000	362 hundred	361 views 644 subs
